# Supplementary material for: Are carbon emissions trading and green financial instruments synergistic? -Comprehensive quantitative research based on content analysis
Source: PLoS One. 2024 Mar 7;19(3):e0298601. doi: 10.1371/journal.pone.0298601 (PMC10919601; doi:10.1371/journal.pone.0298601)
Supplement: S1 Data — (ZIP) [file pone.0298601.s001.zip › Data/地级市Z_F工作报告.zip!/╜¡╬≈/╥╦┤║╩╨/└┤╫╘╣1⁄2╓┌║┼í╛┬φ┐╦╩2╛▌═°í┐_2011.docx]

各位代表：

现在，我代表市人民政府向大会报告政府工作，并就《宜春市国民经济和社会发展第十二个五年规划纲要（草案）》作出说明，请各位代表审议，各位市政协委员和列席会议的同志提出意见。

一、2010年工作简要回顾

2010年，全市人民在省委、省政府和市委的正确领导下，围绕“止后移、争进位、站前列”的总体目标，深入贯彻落实科学发展观，积极应对各种严峻挑战和考验，齐心协力，攻坚克难，锐意进取，完成了市二届人大五次会议确定的主要任务。

--综合实力明显提升。全市生产总值突破800亿元，达870亿元，比上年增长14.1%；财政总收入跨越百亿元，达107.9亿元，增长33.9%，其中，地方财政收入66.3亿元，增长39.1%；市本级财政总收入首次突破10亿元大关，财政总收入超10亿元的县（市、区）数量达到4个，占全省的五分之一；丰城跨入“全国百强县”行列；樟树进入“中国产业发展能力百强县市”。全市规模以上工业增加值288.6亿元，增长24.9%。全社会固定资产投资665.9亿元，增长31.9%。

--经济结构不断优化。三次产业结构比例调整为19.3：56.5：24.2，非农产业比重首次突破80%，尤其工业主导地位更为凸显。财政总收入占GDP的比重达12.4%，同比提高0.9个百分点；税收收入占财政总收入的比重达90.6%，居全省前列。全市民营经济实现税收69.2亿元，增长36%，占全市税收总额的比重达70.8%。十大产业基地完成主营业务收入464.78亿元，增长43.8%；实现利税57.51亿元，增长43.5%。全市工业园区主营业务收入跨越1000亿元大关，达1044.4亿元，增长49.3%，其中过百亿元的工业园区达6个，占全省的五分之一。

--项目建设取得突破。全年引进亿元以上项目116个，其中5亿元以上项目31个，总投资超过200亿元，项目数量连续两年居全省之首。富士康成功落户丰城，实现引进世界500强零的突破。丰城华伍在深圳创业板成功上市。靖安洪屏抽水蓄能电站、高安陶瓷产业总部经济城顺利开工，明月山机场可研报告获国家发改委正式批复，有望明年建成通航。昌铜高速、杭南长高铁开工建设，高安建陶基地和樟树盐化工基地铁路专用线建设进展顺利，咸宜井铁路列入省、部“十二五”铁路建设规划。公路“迎国检”工作圆满完成。

--体制改革扎实推进。国企改革取得重大突破，238户国有工业企业改革全面完成；14户省属企业完成社区移交；164户非工国有、集体企业改革基本完成，改革进度走在全省前列。医药卫生体制改革全面加快，五项重点工作取得明显成效，基本医疗保障制度覆盖面不断扩大，新型农村合作医疗参合率达97.3%，提前完成省下达任务。“两集中、两到位”改革成果继续巩固，“一站式”服务功能进一步增强，行政服务效能有效提升，公共资源交易依法有序推进，涉企收费“三零两减半”政策全面落实，共为90家企业减免收费870多万元。政府机构改革全面完成。文化体制改革深入推进。

--开放水平稳步提高。实际利用外资3.6亿美元，增长10.9%；完成外贸出口5.57亿美元，增长68.8%；引进省外资金193.4亿元，增长43.2%，其中亿元以上工业项目98个，5000万元以上省外资金工业项目145个，项目个数列全省第一；成功争得“全国加工贸易梯度转移重点承接地”品牌。深入开展“锂能产业招商突击月”和“锂能产业基地建设百日攻坚战”活动，全市锂能产业签约项目80个，10亿元以上项目18个，签约资金800余亿元，首期投资金额218亿元，已进资、动工或投产企业28家，其中3家企业生产出锂电产品、4家企业生产出锂电池样车。

--生态建设成效显著。造林绿化“一大四小”工程超额完成任务，居全省综合先进市前列，樟树、丰城等6个县（市）获得表彰。森林防火和林改工作成果进一步巩固，被评为“全国森林防火工作先进市”；宜丰荣获“全国林改典型县”。中心城“13579”绿色工程纵深推进。农村清洁工程开展试点，袁州等8个县（市、区）获省表彰。土地整理项目和“造地增粮富民”工程大力实施，新增耕地1.75万亩，连续11年实现耕地总量动态平衡，基本农田保护率稳定在85.5%以上。城市污水处理厂全面投入使用，中心城污水处理率达86%；各县（市）城市污水处理厂运行基本稳定达标。4个工业园区列入省级生态园区创建试点。全省生态园林城市创建实现“一次申报、率先摘牌”，增获“中国宜居城市”、“人民满意城市”、“中国最佳休闲养生城市”等多张国家级名片。

--抗洪救灾全面胜利。全区域遭遇特大洪涝灾害，降雨之强、险情之多、灾害之重均属历史罕见。危难关头，各级众志成城，科学应对，全力以赴防大汛、抗大洪、抢大险、救大灾，成功抢险93座水库、化解970处圩堤险情，紧急转移威胁区群众13.7万人，最大限度地保护了受灾群众的生命财产安全。洪灾过后，积极开展生产自救，全面打响水利建设主攻战，赢得了抗洪斗争的重大胜利和大灾之年的农业丰收，得到省委、省政府和广大群众的充分肯定。

--社会事业和谐共进。教育“三名工程”稳步实施，素质教育全面推进，全市高考二本以上上线人数首次突破万人，宜春实验中学等3个单位荣获“全国学校艺术教育工作先进单位”。在省政府对县级政府教育工作督导评估中，丰城获“全省教育工作先进县”，奉新受到通报表扬。成功承办 “欢乐中国行·魅力宜春”大型文艺晚会、第23届全国摄影艺术展等重大活动，上高农民摄影展获全国摄影群星奖，宜春文化艺术中心落成并成功举办中国爱乐乐团交响音乐会等重大演艺活动。樟树被命名为“中国剪纸艺术之乡”。第十三届省运会上，我市奖牌、金牌和总分均居全省第二。《王大茶油》、《春满明月山》分别获得全国小戏小品大赛优秀剧目奖和第二届中国秧歌节最高奖。《宜春市志》作为新中国成立以来宜春的第一部地方志顺利出版发行。人口计生工作成效显著，荣获“全国人口计划生育综合改革示范市”。

--民生保障有效落实。全市民生支出突破120亿元，占财政总支出的七成以上。全面完成省政府下达的民生工程8大方面、88项指标，其中64项超额完成。全市2532个行政村100%实现通水泥（油）路、81个渡口100%架通桥梁。城镇新增就业、新增转移农村劳动力、新农保参保、小额贷款发放、残疾人基层组织建设等工作走在全省前列。免费治疗白内障患者9103例，完成网络直报8259例，居全省第一。袁州在全省率先启动195座小（二）型病险水库溢洪道达标建设。保障性住房建设力度加大，新开工面积148.06万平方米，新建住房18895套(户)。市场价格监管加强，确保了群众基本生活。城镇居民人均可支配收入和农民人均纯收入分别达到14333元、5799元，分别增长10.2%和 14.2 %。

--文明创建成果丰硕。宜春中心城、万载、宜丰荣获“省文明城市”称号，103个单位获“省文明单位”荣誉。被国家主流媒体誉为宜春英模群体“井喷现象”，成为我市新时代的闪光“名片”，王茂华、谭良才被评为“全国见义勇为英雄”、“全省见义勇为标兵”，我市被评为“全国见义勇为优秀城市”；32人先后入选“中国好人榜”，人数居全国设区市首位。农村和谐文化建设“一村一品”活动深入推进，涌现出100多个各具特色的和谐文化示范村，农家书屋覆盖行政村率达42.5%。

回顾过去的一年，我们在政府工作中坚持了“五个注重”：

（一）注重发展速度与增长质量的统一。坚持以加快转变发展方式为抓手，以促进产业优化升级为重点，着力提高经济发展质量和内生动力。全市规模以上工业完成主营业务收入1227.46亿元，增长44.1%；利税总额160.76亿元，增长59%；工业经济效益综合指数275.9%，提高36.9个百分点；工业用电量64.4亿千瓦时，增长32.1%，高出全省平均水平12.9个百分点。产业支撑能力明显增强，电力能源、机电、食品、建材和医药等五大支柱产业完成工业增加值188.11亿元，增长26.8%，占规模以上工业的65.2%。围绕“决战园区、跨越千亿”目标，大力实施工业强攻战，滚动实施了157个亿元以上工业项目，其中新开工亿元以上项目74个。工业投资增长40%，占全市固定资产投资的74%。全省工业流动现场会在宜春召开，对我市以新型工业化为导向的经济增长方式予以肯定。扎实推进科技创新“六个一”工程，仁和集团中药集成创新技术平台获国家科技重大专项立项支持，江西惟思特等4个项目获省政府高新技术产业重大项目，江西福斯特和联威新能源等5个项目获省科技重大专项支持，江特电机研发平台被认定为省特种电机工程技术研究中心。全市培育省级以上创新型企业16家，新批高新技术企业11家，开发省级重点新产品146个。市、县两级先后与26所高校、科研院所建立科技合作关系，产学研科技合作项目达170余项。

（二）注重做大市本级与做强县市区的并重。坚持实施重大项目推动市本级、带动县市区发展战略，围绕区域性产业特色和优势资源，深入开展了重大产业与重点项目推进年活动，一批重大项目相继落户。市、县两级共争取国家和省项目资金25.64亿元，增长28%，其中无偿资金15.64亿元；列入省重大项目调度会的项目23个，争取省级重大项目用地指标2.34万亩。积极推进市本级“三大战役”，市本级财政总收入达10.65亿元，增长40.2%；中心城区六大产业引进落户投资3000万元以上项目146个，签约资金800.3亿元，实际进资项目86个。在做大做强市本级的同时，强力启动县域经济发展三年大竞赛，加强对再创新一轮县域经济新优势的支持和指导。各县（市、区）自我加压、发奋努力，综合实力均上新台阶， GDP大幅增长；固定资产投资大量落地；丰城、樟树财政总收入分别高达27.6亿元、17.1亿元，高安、袁州均突破10亿元，上高超8亿元，过亿元乡镇（街道）达到8个，比上年增加3个。更加注重市本级与县市区的联动发展，全市接待游客突破800万人次，增长24.5%；旅游总收入突破60亿元，增长28.5%。全市银行机构资产总额首次突破1000亿元，新增信贷规模再次突破100亿元。南昌银行、九江银行、顺银村镇银行在宜春开业；浦发银行、上饶银行初定宜春落户；宜春农村商业银行组建工作顺利推进。批复筹建小额贷款公司6家，占全省的四分之一，总数跃居全省第四。樟树被评为“2010年中国最具特色金融生态示范城市”。

（三）注重城市建设与“三农”工作的统筹。以实施城建三年大会战为契机，全力推进新型城镇化建设，城市基础设施日益完善，城镇化率达41.1%。中心城区城市建设管理暗访连续6年排名全省第一。全市城建大会战开工项目400余个，完成投资600多亿元，其中基础设施项目完成267亿元。中心城区启动实施了“六个六”工程和166个城建项目，已开工88个，基本完工15个，完成投资102亿元。人口倍增工作稳步推进，中心城区人口达47.6万。各地城镇化进程进一步加快，县（市）城市框架全面拉开。农业产业化取得新突破，市级以上农业产业化龙头企业发展到176家，其中国家级4家、省级52家，列全省第三位。农民专业合作社突破1000家，达到1226家。全国水稻新品种展示现场观摩会、全国农村五保供养工作会、全省农业产业化现场会和农民专业合作社建设与发展经验交流会分别在奉新、丰城和上高召开。有机农产品数量和基地面积稳步增加，全市有机农产品认证71个，基地面积49.34万亩，丰城等3个市（县）荣获“全国粮食生产先进市（县）”、丰城被授予全国唯一的高产油茶之乡；万载成为全省唯一的有机（绿色）食品产业基地。获批国家级农业标准化示范区12个、省级现代农业示范区3个。加快推进新农村建设，891个村点全部制定产业发展规划，形成“一村一品”产业村691个。

（四）注重打造金山银山与保护绿水青山的兼顾。坚持生态立市战略，着力打造中部地区最佳宜居城市。在确保赣江水质、宜春中心城空气质量自动监测站稳定运行的同时，全面启动了袁河3个水质自动监测站和宜阳新区1个空气质量自动监测子站建设，积极做好各县（市、区）集中饮用水源和7条河流控断面水质监测，确保了水质全年优于三类，城区空气质量全年稳定达到二级标准。深入开展专项污染整治，加快落后产能淘汰，查处违法排污企业43家。顺利通过省政府节能目标责任考核，丰城资源循环利用产业基地被授予“全国工业固废综合利用示范基地”，丰城被列为“全省循环经济试点县（市）”，宜春经济开发区等3个园区被列为“全省循环经济试点工业园区”，宏宇能源等4家企业被列为“全省循环经济试点企业”，上高荣获“国家绿色能源示范县”称号。大力推广节能新产品和新技术，宏宇能源和合力照明等企业产品列入江西省节能产品推荐目录。以“国家现代林业示范市”和“森林宜春”建设为抓手，大力实施造林绿化，完成植树造林49万亩、通道绿化2054.6公里。袁州在全省率先整区启动封山育林。全市新增省级森林公园10个、城市森林公园5个。

（五）注重改革攻坚与和谐稳定的协调。全面深化各项改革，有效激发内生发展动力。工业企业改革全面完成，七大系统国企改革顺利推进。财政改革取得新成效，部门综合预算管理全面推行，市本级公务卡改革试点工作稳步实施，政府采购“管采分离”更加规范，纳税信息一体化管理体系进一步完善。不断加大民生投入，健全社会保障体系，促进和谐稳定。相继开展“万名干部下基层、和谐稳定进乡村”集中行动月和“为市民解忧、促城区和谐”专项活动，取得了亲民惠民促和谐的重要成果。积极探索维稳长效机制建设，制定下发了《关于建立完善长效管理机制创建城乡和谐平安新秩序的决定》等“1＋4”配套文件，促进群众工作经常化、制度化。切实抓好上海世博会和广州亚运会、亚残运会期间的维稳工作，发挥了“护城河”的作用。开展了以打击“两抢一盗”为重点的专项整治行动，社会治安进一步好转，公众安全感继续保持全省前列。我市转变执法理念、改进执法方式工作和丰城社会管理创新的经验，得到中央肯定。安全生产保持稳定向上的良好态势。

与此同时，切实加强政府自身建设，不断提高施政水平。坚持依法行政，加快政府职能转变，自觉接受人大法律监督和政协民主监督，认真听取各方面的意见和建议，推进决策科学化和民主化，办理人大代表建议104件、政协委员提案180件。加强政府法制建设，大力推进规范性文件清理，加大执法监督力度，向省政府和市人大常委会报送规范性文件备案5件。“五五普法”顺利结束，荣获“全国普法依法治理先进单位”。进一步拓宽政务公开和政府信息公开渠道，积极搭建网络问政平台，组织“在线访谈”11期。深入开展“创业服务年”活动，抓好党风廉政建设责任制的落实，推进惩治和预防腐败体系建设，大力发挥效能监察和审计监督作用，切实整治工程建设领域和损害群众利益的突出问题，政务环境进一步优化。国防后备力量和民兵预备役建设、人民防空、国家安全、新闻外宣、外事侨务、民族宗教、涉台事务、气象服务、防震减灾、妇女儿童、统计、档案、扶贫、老龄、残疾人和红十字会等各项工作都取得了新进步。

各位代表，过去的一年，是宜春发展进程中极不平凡的一年。全市人民迎难奋进，团结拼搏，实现了经济社会全面发展的目标，并超额完成了“十一五”规划的任务，24个主要经济指标中有12个指标提前一年完成，其中生产总值、财政收入、固定资产投资、社会消费品零售总额翻了一番多，规模以上工业增加值、外贸出口翻了两番多。五年来，综合实力实现大跨越。生产总值年均递增13.6%，人均生产总值突破2000美元；财政总收入年均递增28.2%，全社会固定资产投资年均递增32%，规模以上工业增加值年均递增25%，社会消费品零售总额年均递增18.3%。城乡面貌发生大变化。建成区面积由2005年的129平方公里扩大到207平方公里，城镇化率年均提高1.6个百分点。人居环境持续改善，城市品位极大提升。武吉高速宜春段等一批高等级公路建成通车，明月山机场等重点工程开工建设，基本实现村村通水泥（油）路，综合交通网络初步形成。社会事业取得大进步。城乡免费义务教育全面实施，基础教育得到巩固，高等教育发展加快。科技创新能力不断提升。计划生育基本国策有效落实。生活水平有了大提高。城镇居民人均可支配收入年均递增11.7%；农民人均纯收入年均递增11.1%，是新中国成立以来城乡居民收入增幅最大的五年。居住条件不断改善，治安秩序进一步好转，城乡居民对治安的满意度连续5年保持在95%以上，连续5年居全省前列。生态环境持续大改善。生态环境质量继续保持全省领先水平。全市生活饮用水水源地水质和主要河流地表水质量达到国家规定标准。中心城空气质量达到国家二级标准。节能减排完成“十一五”预期目标。发展的实绩表明，“十一五”时期是我市综合实力快速提升的五年，是城乡面貌日新月异的五年，是人民生活显著改善的五年，是发展活力不断增强的五年。成绩的取得，得益于省委、省政府和市委的坚强领导，离不开市人大、市政协的监督支持，凝聚着全市人民的辛勤汗水。在此，我代表市人民政府，向全市广大工人、农民、知识分子、干部和历任老领导、老同志，向各民主党派、工商联、无党派和社会各界人士，向驻宜部队、武警官兵、公安干警、中央和省驻宜单位，致以崇高的敬意！向所有关心、支持宜春发展的社会各界朋友和海内外友好人士，表示衷心的感谢！

在看到成绩的同时，我们也清醒地认识到，全市经济社会发展仍面临诸多困难和问题：经济总量偏小、产业聚集度偏低，转变发展方式的任务还很艰巨；资源环境约束不断加大，加快发展与加速转型的矛盾增多；区域发展不平衡，中心城凝聚与辐射功能不强，农民持续增收和统筹城乡发展的难度增加；部分群众生活比较困难，社会不和谐因素和安全维稳的压力依然较大；政府自身建设仍需加强，依法行政和工作效能有待进一步提高，等等。对这些困难和问题，我们将更加认真、有力地加以解决。

二、关于“十二五”规划纲要（草案）的说明

（一）总体发展目标。围绕 “决战‘十二五’、奋力站前列”的总体要求，以加快建设 “亚洲锂都、宜居城市、森林宜春、月亮之都”为目标，在综合分析各方面因素的基础上，《宜春市国民经济和社会发展第十二个五年规划纲要（草案）》（以下简称《纲要（草案）》）提出了“十二五”时期我市经济社会发展的主要目标，包括经济发展、结构调整、社会发展、民生改善、环境保护五大类、38个主要指标，其中，生产总值年均增长12%以上，达到1750亿元，力争突破2000亿元；财政总收入年均增长18%，达到247亿元，力争突破300亿元；全社会固定资产投资年均增长22%，达到1800亿元，力争突破2000亿元；城镇居民人均可支配收入、农民人均纯收入年均增长11%，分别达到24150元、9770元。这些目标体现了进位赶超、跨越发展的要求，体现了转型升级、协调发展的要求，体现了惠及民生、和谐发展的要求，体现了绿色崛起、可持续发展的要求。

（二）主要任务措施

围绕上述目标任务，《纲要（草案）》提出了全市今后五年经济社会发展的九项主要措施。

1、推进新型工业化，构建现代产业体系。围绕“扩规模、调结构、转方式、促发展”的思路，按照区域协作、优势强化、分类引导的原则，重点培育锂电新能源、建筑陶瓷、生物医药、机械电子、纺织服装鞋革、食品、竹加工等七大产业集群。突出做大做强产业链、培育大产业，重点培育盐化工、烟花爆竹、新型建材、资源循环利用、货运专用车等特色产业基地。

2、推进农业农村现代化，建设社会主义新农村。着力推进农业发展方式的转变，加快构建高产、优质、高效、生态、安全的现代农业体系。全面推进十大优势特色产业发展，重点发展油茶、毛竹、粮食、畜禽和有机（富硒）农产品五大产业。抓好农田水利、农业服务体系、农业防灾减灾设施建设。继续把繁荣农村经济作为新农村建设首要任务、把改善居住环境作为新农村建设的突破口，进一步优化产业结构、优化村庄布局、美化农村环境。

3、统筹区域协调发展，加快新型城镇化建设。立足产业基础和生态优势，将“打造全省生态建设的示范区、低碳经济和低碳产业的崛起区、宜居城市建设的先行区”作为中心城发展定位。构建以宜万组合和丰樟高组合为区域中心、5个县城为次中心、小城镇为重要节点的城镇体系。把发展县域经济作为统筹城乡、加快崛起的重要抓手，促进县域发挥各自优势，壮大经济实力，实现跨越发展。

4、加快发展服务业，提升社会化服务水平。按照“提升传统服务业，发展现代服务业，做强有比较优势服务业”的总体思路，重点发展旅游业、商贸物流业、金融业、社区服务业和信息产业等现代服务业。把发展服务业作为加快产业结构调整、转变经济发展方式、完善城市服务功能、实现全面协调可持续发展的重要途径，努力将宜春打造成覆盖赣西及周边地区城市群的服务业高地、全国著名的养生休闲度假旅游胜地。

5、实施科技创新和人才强市战略，增强区域竞争力。以创新型宜春建设为抓手，积极探索科技发展新模式，实施五大科技工程，充分发挥科技工作在转变经济发展方式、经济结构转型和产业技术升级中的作用。充分发挥政府在发展教育中的主导和管理作用，努力建立结构合理、质量优良、公办民办并举的多元化教育发展体系。积极实施“6666”人才工程，壮大人才队伍，提高人才竞争力，加快构建区域性人才高地。

6、深化体制机制改革，扩大对内对外开放。把握改革的节奏、力度和时机，谋划和推动重点领域、关键环节的改革攻坚，解决束缚发展的深层次矛盾和问题，为科学发展、进位赶超提供体制机制保障和最优发展环境。紧紧抓住经济全球化和世界经济格局调整的战略机遇，充分利用国际国内两个市场、两种资源，在更广领域、更高层次上对外开放，为经济社会发展提供更加广阔的空间和持久的动力。

7、统筹社会各项事业，实现全面协调发展。以实施《宜春市文化发展纲要》为抓手，全面实施“三大文化”工程，着力提高文化产业在经济总量中的比重。加强公共体育设施建设，积极发展竞技体育，加快体育产业发展。加强城乡医疗卫生服务能力建设，加快医药卫生信息系统建设，巩固和发展新型农村合作医疗、城镇职工和居民医疗保险制度。建立和完善食品药品安全监管机制。

8、保障和改善民生，维护社会和谐稳定。以构建社会主义和谐社会为根本要求，坚持民生优先、公平正义、成果共享，着力解决影响社会和谐稳定的源头性、基础性、根本性问题，促进全体居民共享与经济发展水平相适应的基本公共服务，促进经济社会协调发展、良性互动。

9、加强基础设施建设，提高发展保障能力。围绕公路扩网、铁路扩能、水运发展、机场建设，形成快速便捷的综合立体交通运输网络。优先发展低碳能源和传统能源，积极开发利用新能源和可再生能源，推进电力需求管理。继续加强防洪减灾工程体系建设，加强大中型灌区续建配套，全面完成小型水库除险加固任务。合理开发和优化配置水资源，加强水资源保护。加强城镇污水处理基础设施建设，加大农村环境整治力度，做好主要污染物总量减排工作。

三、关于2011年政府工作安排

2011年是“十二五”的开局之年，是建党90周年。今年政府工作的总体要求是：认真贯彻党的十七届五中全会、中央经济工作会议和省委十二届十四次全体会议精神，以邓小平理论和“三个代表”重要思想为指导，以科学发展为主题，以加快转变经济发展方式为主线，以推进工业三年强攻战、城建三年大会战、农业三年升级战、县域经济三年大竞赛为抓手，以开展“产业升级年”活动为动力，着力扩大固定资产投资总量，着力提升产业发展水平，着力增加人民群众幸福指数，着力推进社会和谐稳定，实现发展速度再创新高、发展质量再上台阶、区域经济再增特色，努力实现“十二五”经济社会发展开门红。

综合考虑各种因素，今年国民经济和社会发展主要目标建议为：生产总值增长13%；财政总收入增长19%，其中地方财政收入增长19%；规模以上工业增加值增长25%；全社会固定资产投资增长25%；社会消费品零售总额增长18%；引进省外5000万元以上工业项目资金增长15%；实际利用外资增长10%；外贸出口增长20%；城镇化率提高2个百分点；农民人均纯收入增长11%，城镇居民人均可支配收入增长11 %；城镇登记失业率控制在4.5%以内，人口自然增长率控制在8‰以内。

为实现上述目标，将重点抓好以下六方面工作：

(一)加快推进产业集聚和项目建设，切实增强经济发展的协调性和竞争力

千方百计引项目。继续实施重大产业招商，充分利用我市“全国加工贸易梯度转移重点承接地”这一品牌优势，突出重点区域，盯紧重点产业，深化“敲千家门、引万名商”活动，着力引进一批规模大、带动能力强、科技水平高的龙头骨干企业，进一步加速产业集聚升级，力争全年引进亿元或千万美元以上重点项目150个，5亿元以上项目35个，引资总额达到250亿元。突出加强与中央、省属国有大型企业、外资企业尤其是世界500强、国内200强、行业50强企业的对接，努力在招大引强上实现新的重大突破。继续坚持市直单位招商，完善考核办法，切实兑现奖惩，力争市直单位全年引进项目100个，亿元或千万美元以上重点项目30个，引资总额80亿元。进一步健全重大项目协调推进机制，完善责任主体，凝聚部门合力，促进在谈项目早落户、已批项目早开工、建成项目早见效。大力推进口岸平台建设，加快宜春锂电铁路口岸作业区、高安陶瓷基地铁路口岸作业区、高安国家陶瓷质量检验检测中心、万载烟花爆竹公路口岸作业区建设，力争在宜丰设立保税物流中心，协调开通万载烟花爆竹经上海出口运输通道，进一步降低企业物流成本。

全力以赴争项目。继续将跑项争资作为经济工作的重中之重来谋划和落实，敏锐把握政策实施的力度和节奏，重点围绕“十二五”规划和战略性新兴产业挖掘项目，围绕优势资源开发、民生需求培育项目，围绕中部地区崛起战略和鄱阳湖生态经济区建设争取项目，力争一批带动力强、成长性好的项目进入国家、省规划“笼子”。加强与国家、省相关部门的沟通联系，建立完善部门联动机制，形成工作合力，尤其要充分利用我市国家级锂能基地的金字招牌，千方百计挤进“十城千辆”、新能源汽车基地和全国低碳试点城市，力争建立省级、国家级锂电产品质量监督检测中心、测试中心和研发中心。

争分夺秒促项目。按照“项目化、时间表、责任人”的要求，进一步完善跟踪服务机制，加快推进重点工程建设。力争完成明月跨铁路大桥建设，推进明月山机场、明月大道、宜春综合交通枢纽建设。积极配合杭南长、昌吉赣客运专线建设。完成昌铜高速奉新-南昌段建设，推进昌铜高速奉新-铜鼓段建设，力争启动宜丰-上高-新余一级公路改造，推进宜春-万载-铜鼓高速公路前期工作，力争宜春-万载段尽早开工。支持昌樟高速扩建、丰城铁路专线建设。启动高安建陶基地、樟树盐化工铁路专线运营。继续推进洪屏抽水蓄能电站、西气东输管道和丰龙煤矿建设。抓紧做好咸宜井铁路、丰电三期、万载热电联产、上高电厂和铜鼓、上高风电等项目前期工作。尽快启动宜丰220千伏输变电工程、宜阳新区输变电工程建设，力争2011年竣工投运；启动袁州彬江110千伏、宜春锂能产业基地220千伏、110千伏输变电和10千伏配电工程建设。继续实施优质粮食产业工程，加快大中型泵站更新改造，推进潦河、锦北、丰东、袁北灌区配套和节水改造工程。抓好市、县生活污水处理厂及垃圾无害化处理工程建设，加快工业园区污水处理厂及配套管网建设，做好中心城区及县（市）应急水源建设的规划论证。

（二）着力提升大竞赛的成效和水平，争创县域经济新优势

深入推进工业强攻战。围绕“全市规模以上工业企业主营业务收入决战1600亿，工业园区主营业务收入实现1400亿”的总体目标，注重做到“三个坚持”：一是坚持把新兴产业作为壮大工业总量的增长极。继续举全市之力，有效整合锂矿资源，强力推进锂能产业发展。大力推进动力电池的研发生产，突出整车制造，争取产业链每个环节拥有5-8个项目，尽快形成产业集聚，做响“亚洲锂都”品牌。二是坚持把产业基地作为提高工业承载能力的主战场。进一步完善产业规划，强化发展举措，以龙头骨干企业的支撑和带动，加速形成完整的产业体系。十大产业基地主营业务收入达到680亿元，利税总额达到80亿元，力争1-2个基地进入国家产业示范基地行列。尤其把工业园区成规模、上水平、见效益作为着力点，力争2个以上挤进全省第一方阵。以争创国家级开发区为动力，加大市直部门支持力度，确保宜春经济开发区主营业务收入突破150亿元，财政收入突破10亿元。三是坚持把重大项目作为增强工业发展后劲的支撑力。加快培育壮大一批重点骨干企业，拉长产业链，提高附加值。按照“上下延伸、协作配套”的要求，在促进大产品、大项目落户的同时，切实加强部件产品项目的跟踪推进。实施滚动淘汰制，精心筛选100户优势企业，实行市、县两级重点调度、重点支持和重点奖励。力争规模以上工业企业达到1000户，主营业务收入超10亿元企业达到15户，超5亿元企业达到50户，超亿元企业达到400户。全力推进一批投资10亿元以上重大项目建设，重点抓好联威锂电、宏宇能源、江西惟思特、华信手机、四特“两城”、泓邦超高压电缆、奉新纺织印染集控区等项目的推进建设；抓紧青山能源、比亚迪等项目的落地开工；加快宜春重工、泰明光伏、格林美电子废弃物回收利用等项目的竣工投产；促进斯米克陶瓷、龙工齿轮等项目的达产达标，加快形成一批工业新增长点。

深入推进城建大会战。加快宜春中心城区“六个六”工程实施。争取未开工项目全部开工。全面启动“五区”建设，突出抓好宜阳新区核心区建设，全面提升城市承载能力。进一步加大城市创建力度，力争2013年在全省率先获得“国家生态园林城市”称号。抓紧做好“13579”绿色工程收尾工作，大力开展全民义务植树，继续谋划实施一批高品位的园林绿化建设工程。大力实施“数字城管”工程，不断提高城市管理精细化水平。加快县（市）城市建设提质扩容。按照“三年拉框架，再造新城区”的总体要求，进一步加大城市投入，突出抓好城市基础设施和公共服务设施建设。推进丰城、樟树、高安三市城市要素集聚，扩大城市规模，加快构建中等城市基本框架，努力打造市域区位中心城市；加快其他各县建设步伐，进一步完善城市功能和配套设施。继续开展生态县、生态乡镇和生态村创建，做好靖安争取国家级生态县等申报工作，力争宜丰、铜鼓两县命名为省级生态县，全市5个乡镇、6个村命名为国家级生态乡镇和生态村，新增省级生态乡镇26个、省级生态村20个。进一步加强污水处理厂运行监管。加快省级示范镇和市级重点镇建设。强化村镇规划管理，依法管好农村用地，加快小城镇建设。以示范镇和重点镇建设为龙头，着力培育一批工业强镇、商贸大镇、旅游名镇和全省百强镇，不断拓展新型城镇化发展空间。大力开展道路街巷、违章搭建、临街建筑、环境卫生、集贸市场“五整治”和园林绿化、公用设施、景观环境“三建设”达标活动，使全市集镇环境有较大改善。

深入推进农业产业化升级战。大力提升农业产业化水平。继续推进农业产业招商，着力引进一批实力强、影响大、品牌硬、辐射带动能力强的龙头企业，确保农业招商引资额达到招商引资总额的20%以上。重点加快油茶、毛竹、粮食、畜禽和有机（富硒）农产品等五大产业发展，力争五大产业综合产值突破300亿元，年销售收入亿元以上龙头企业突破40家。按照民办、民管、民受益和利益共享、风险共担的原则，加快推进农民专业合作社建设，力争全市农民专业合作社增至1600家以上。积极实施品牌提升工程，大力扶持龙头企业、农民专业合作社创建省部级名牌农产品，争创宜春农业知名品牌。加快现代农业示范区建设。突出抓好现有的12个国家级农业标准化示范区和3个省级现代农业示范区建设，力争再创国家级现代农业示范区1个、省级现代农业示范区2个以上。加快推进农业机械化步伐，力争农机总动力突破700万千瓦。抓好“菜篮子”工程建设，力争今年中心城区蔬菜基地面积达1.3万亩，三年内达到2万亩，蔬菜自给率达80%。落实地方储备粮任务，确保国家粮食安全。继续推进新农村建设。大力实施造林绿化“一大四小”工程和农村清洁工程，突出抓好通道绿化等“五大提升工程”，确保完成造林40万亩，力争 “一大四小”工程建设继续在全省站前列。积极探索和完善新农村建设村镇联动、联村整片推进模式，突出抓好20个村镇联动试点，100个整村推进示范点，4000个集镇和自然村的农村垃圾无害化处理。扎实搞好农田水利基本建设。深入开展“农田水利建设年”活动，重点抓好5座重点圩堤的应急处理和5条中小河流的治理，抓紧135座小（一）型水库除险加固、1358座小（二）型病险水库溢洪道达标建设和大坝清杂、270座小（二）型病险水库应急除险，做好水毁工程修复工作，确保防洪安全。抓好农村饮水安全工程建设，解决22万人饮水安全问题。

（三）加快转变经济发展方式，进一步推进产业结构优化升级

大力促进科技创新。推进科技创新“六个一”工程，加快锂电新能源、生物和新医药、机电、新材料、现代农业和有机食品等高新技术产业发展，确保高新技术产业增加值达到60亿元，增长20%以上。深入开展“科技入园、科技进村、科技惠民”活动，进一步加强科研机构和科技人才建设，建立和完善一批重点实验室或工程技术研究中心，大力扶持科技创新团队建设。积极申报全国节能试点城市和一批以锂电节能为主的国家科技支撑项目。着力深化人事制度改革，加强公务员队伍建设，继续实施“6666”人才工程，大力引进高层次急需人才，为经济社会发展提供人才支撑。加快科技成果的培育和引进，加强科技合作交流，深入推进产学研合作，推动与中南大学、同济大学等院校的战略合作，不断提高科技创新水平。认真抓好节能减排，严格落实环境保护、节能减排各项措施，积极推广节能减排新技术，加快实施节能减排重点工程，强化环境监察与目标管理，坚决打击环境违法行为。

加快推进服务业发展。把服务业作为扩大消费、调整结构的重要着力点，进一步加强资源整合，加大政策扶持，强化产业集聚，高起点、高标准规划建设一批服务业重点项目，着力把宜春打造成赣西服务业新高地和湘赣边际商贸休闲中心。全力推进中心城区五金商贸城、龙达物流、大润发超市、汇金商贸广场、赣西农产品批发市场三期扩建等项目建设。积极推进红林大酒店、汇龙大酒店等五星级宾馆建设。不断完善中介服务市场，促进中介行业健康发展，大力培育服务外包、会展等新兴服务业态，带动生产性服务业整体水平的提高。加强对总部经济的研究，明确发展思路，制定发展规划，出台优惠政策，采取“内强外引”的办法，以丰城龙津湖总部经济基地、高安陶瓷产业基地总部经济城为示范，依托明月山独特的山水资源优势，加快启动明月山总部经济基地建设，积极引进一批国内、省内知名企业入驻宜春设立地区总部或分支机构，促进总部经济快速发展。

努力扩大消费需求。继续打好以明月山温泉风景区为龙头的旅游升级战，突出“养生休闲度假旅游胜地”总体定位，打响“月亮之都、禅宗圣地”品牌。精心做好第五届月亮文化节、第六届中国竹文化节等重大节庆活动的筹备工作，加快禅都文化博览园、宜春国际温泉禅修中心、明月山温泉康疗中心、梅花国际温泉度假区、袁州酌江风景区二期、樟树新恒基养生天堂和閤皂山道教休闲度假区、丰城仙姑岭风景区、铜鼓温泉旅游休闲度假区、靖安中部梦幻城、宜丰东方禅文化园、奉新百丈寺与仰山漂流等一批旅游重大项目建设，完善配套服务功能，促进旅游全面升级，力争年接待游客突破1000万人次，旅游总收入突破80亿元。继续开展“六个十佳”创评工作，力争启动明月山温泉风景区创新中国成立家5A级景区，推进铜鼓红色旅游景区和奉新百丈山景区创新中国成立家4A级景区，争取奉新宋应星公园、高安贾家古村和宜丰东方禅文化园创新中国成立家3A级景区。扎实推进“万村千乡市场工程”和“新农村现代流通网络工程”建设，加快农村便利连锁店、放心店和商品配送中心建设，继续推进家电、汽车、摩托车下乡以及家电、汽车以旧换新工作，促进农村消费升级。扩大和完善消费信贷，做好基本生活必需品的生产、调运、储备和供给，加强消费市场监管，保持物价稳定，努力营造良好的消费环境。

（四）加快重点领域和关键环节的改革，充分激发经济发展活力

继续推进重点改革。深化国有集体企业改革。按照时间节点要求，力争上半年全面完成纳入考核目标的七个系统的国有企业改革，年底前基本完成纳入考核目标的集体企业改革。加大国有资产监管力度，提高国有资产经营水平。加强政府性债务管理，积极防范和化解财政风险。继续推进医药卫生体制五项重点改革，抓好基本药物制度实施工作，加快公立医院改革，确保城镇职工医保、城镇居民医保参保率及新农合参合率均稳定在92%以上。加快农村改革步伐，稳定农村基本经营制度，在依法自愿有偿的基础上，促进土地适度规模经营和土地承包经营权流转。巩固集体林权制度改革成果，进一步完善林权抵押贷款、森林保险等配套改革。继续深化财税体制改革，强化财政运行监督，规范对民生改善、重点项目等专项资金的管理。

大力激活资本市场。支持各银行机构以扩大信贷规模为重点，多层次、全方位推进银企对接，大力开展信贷营销活动，确保全年新增信贷资金80亿元，力争突破100亿元。继续做好企业融资和再融资工作，扶持正邦化工、远大化工、百神药业等已具备申报上市条件企业，加快上市步伐；大力支持江特、仁和、华伍等已上市公司发展。加大外埠银行引进力度，争取引入1-2家股份制商业银行在宜春设立分支机构。稳妥推进袁州农村信用联社改制，力争上半年完成宜春农村商业银行筹建、年内开业。充分发挥多层次资本市场的融资功能，大力引进风险投资基金、私募基金及金融租赁，务实推进中科招商、深圳国银公司的落户。深入开展融资性担保公司规范整顿，全面推进小额贷款公司组建，确保年底前小额贷款公司机构县域全覆盖。继续推进金融生态环境和社会信用体系建设，扩大银行授信范围和额度，提高融资效率。

加快全民创业步伐。加强创业文化的培育和宣传，完善和落实鼓励全民创业的各项政策措施，扩大市场准入范围，降低市场准入门槛，进一步激发全民创业热情。积极引导有资金、有技术、懂管理的返乡农民工大胆创业；大力支持高校毕业生、下岗失业人员、军队退役士兵自主创业；引导企业强化创业意识，积极开展“二次创业”。大力发展非公有制经济，深入实施中小企业成长工程，遴选一批具有成长潜力的中小企业，优先配置各类生产要素，促其迅速做大做强，力争全市民营经济完成企业增加值700亿元，实现营业收入2000亿元，上交税金90亿元，规模以上民营工业企业对全市民营经济的贡献率达70%以上。

（五）大力保障和改善民生，全面促进社会和谐稳定

积极做好就业和社会保障工作。贯彻实施积极的就业政策，不断拓宽就业渠道，切实解决劳动者求职难、企业用工难等问题。完善落实扶持自主创业政策措施，继续推进首批国家级创业型城市创建。扎实推进社会保险扩面征缴工作，重点抓好改制企业、园区企业、非公有经济员工的养老保险登记、申报工作，妥善做好失业人员、断保职工的续保工作和被征地农民就业培训及生活保障工作。健全城乡居民最低生活保障制度，实现应保尽保，城乡低保月平均保障标准分别提高到300元、130元，农村五保户分散供养、集中供养标准每年分别提高到1560元、2400元。加强城乡困难群众临时救助、医疗救助、法律援助工作，推进残疾人保障体系和服务体系建设。加大保障性住房建设力度，确保全面完成省下达的目标任务。

促进各项社会事业协调发展。全面落实《教育规划纲要》要求，深化教育体制机制改革，促进各级各类教育均衡协调发展。继续推进教育“三名工程”，启动实施义务教育学校标准化建设等10项重大教育工程，重点抓好中心城区5所学校建设，切实解决中小学“大班额”问题。鼓励和支持民办教育，加强民办学校规范管理。全面落实义务教育经费保障机制和教育民生工程，积极解决贫困学生上学难问题。充分发挥宜春学院和宜春职业技术学院服务全市经济社会发展的作用。认真做好国家二类城市语言评估迎检工作，确保顺利通过省政府验收。加强卫生服务能力建设，加快医药卫生信息化步伐，做好疾病预防控制和重大传染病防治工作，确保公共卫生安全。继续实施“光明·微笑”工程，做好儿童白血病免费救治和先天性心脏病普查工作。推进食品药品监督管理体制调整和红十字会体制理顺工作，巩固和完善城乡一体化的食品药品安全综合监管体系建设，大力实施饮食用药安全惠民工程。继续办好明月山写作营。推进国家历史文化名城创建。加快图书馆、文化馆及乡镇综合文化站建设，深入开展农村文化“三项活动”和城市社区文化活动。继续实施农家书屋、职工书屋工程，力争农家书屋覆盖行政村率达76%以上。在宜春中心城和部分县（市）大力推行数字电视，推进“广播村村响”和宽带网络入户进村工程，确保全市70%以上行政村实现“广播村村响”。开展全民健身运动，加强竞技体育工作，推进体育产业向纵深发展，积极承办好第七届全国城运会有关赛事。继续深化人口计划生育综合改革，稳定低生育水平，严格控制出生人口性别比。

高度重视社会安全稳定。认真落实安全生产责任制，强化企业主体责任，健全安全隐患排查治理长效机制，加强基层安监站和监管队伍建设，深入开展重点领域专项整治，坚决防止较大及以上安全生产事故。强化抗震设防措施，提高防震减灾能力。进一步完善应急管理体系，加强综合性应急救援队伍建设，提高应急处理能力。巩固“万名干部下基层、和谐稳定进乡村”活动成效，继续抓好“为市民解忧、促城区和谐”活动，扎实开展“五类信访突出问题”专项治理行动，积极化解信访积案，切实落实维稳“1+4”配套文件，建立健全维稳长效机制，推动各级领导接待群众来访常态化、干部下基层为群众排忧解难常态化。继续加强人民调解、安置帮教、社区矫正和青少年教育管理工作，完善社会治安防控体系与天网工程，创新社会管理，依法防范和打击违法犯罪活动，继续保持群众安全感指数全省领先。

（六）更加注重提升素质和效能，进一步抓好政府自身建设

依法科学理政，大力提升公信力和创造力。深入推进民主法制建设，坚持将依法行政贯穿于政府工作的各个领域和各个环节，努力构建法治政府，确保市、县、乡换届选举工作顺利完成。准确研判分析当前形势，加强对国家和省政策调整的跟踪衔接，用科学的眼光、创新的思路解决前进中的问题。继续完善决策规则和程序，认真执行人大及其常委会的决议、决定，主动接受人大法律监督和政协民主监督。深入推进政务公开，完善政府信息公开和政府新闻发言人制度。

高效务实行政，大力提升执行力和操作力。深入开展“发展提升年”活动，进一步完善政府工作目标责任制、岗位负责制，充分激发政府工作人员的工作热情。加快推进电子政务建设，积极开展网上办公和网上服务。进一步加大行政审批事项清理，完善“两个中心”建设，优化办事程序，提高工作效能，降低行政成本，实现“一个门进场、一条龙服务、一站式办结”。继续做好清项目、优流程、缩时限工作，深入推进“两集中、两到位”改革，全面落实涉企收费“三零两减半”政策。切实精简会议和文件，简化公务接待，把更多的精力用在察实情、出实招、办实事、见实效上。

为民勤政廉政，大力提升亲和力和感召力。坚持问政于民、问需于民、问计于民，深入基层群众，畅开民声通道，办好“在线访谈”，努力使发展目标、工作思路、政策措施更加符合人民群众的愿望和要求。完善为民办实事长效机制，集中力量办好若干件群众最关心、最需要、最急切的实事好事。认真落实党风廉政建设责任制，加强对重点领域、关键环节的廉政监察和审计监督，加大违法违纪案件查处力度，深入开展基层站所政风行风评议活动，以良好的政风行风凝民心、解民忧、惠民生。

各位代表，宜春的发展站在历史新起点，面对时代新使命，科学发展、进位赶超的重任艰巨而光荣。让我们在省委、省政府和市委的领导下，高举中国特色社会主义伟大旗帜，朝着“十二五”发展宏伟蓝图，抢抓机遇，锐意进取，奋力拼搏，为谱写宜春经济社会发展新篇章而努力奋斗！
